# Simulation of the nutritional requirements and energy balance of adult cows in a northern temperate grassland

**DOI:** 10.3389/fvets.2024.1414096

**Published:** 2024-06-19

**Authors:** Tianqi Yu, Ruirui Yan, Xiaoping Xin, Xiaoying Zhang, Guomei Yin

**Affiliations:** ^1^State Key Laboratory of Efficient Utilization of Arid and Semi-Arid Arable Land in Northern China, Institute of Agricultural Resources and Regional Planning, Chinese Academy of Agricultural Sciences, Beijing, China; ^2^Hulun Buir Agricultural Technology Extension Center, Hailar, China; ^3^Grassland Research Institute of Inner Mongolia Academy of Agricultural & Animal Husbandry Sciences, Hohhot, China

**Keywords:** pasture management, energy balance, Hulunbuir grassland, forage-livestock balance, livestock management

## Abstract

The forage-livestock balance is an important component of natural grassland management, and realizing a balance between the nutrient energy demand of domestic animals and the energy supply of grasslands is the core challenge in forage-livestock management. This study was performed at the Xieertala Ranch in Hulunbuir City, Inner Mongolia. Using the GRAZPLAN and GrazFeed models, we examined the forage-livestock energy balance during different grazing periods and physiological stages of livestock growth under natural grazing conditions. Data on pasture conditions, climatic factors, supplemental feeding, and livestock characteristics, were used to analyze the metabolizable energy (ME), metabolizable energy for maintenance (ME_m_), and total metabolizable energy intake (MEI_total_) of grazing livestock. The results showed that the energy balance between forage and animals differed for adult cows at different physiological stages. In the early lactation period, although the MEI_total_ was greater than ME_m_, it did not meet the requirement for ME. MEI_total_ was greater than ME during mid-lactation, but there was still an energy imbalance in the early and late lactation periods. In the late lactation period, MEI_total_ could meet ME requirements from April–September. Adult gestational lactating cows with or without calves were unable to meet their ME requirement, especially in the dry period, even though MEI_total_ was greater than ME_m_. Adult cows at different physiological stages exhibited differences in daily forage intake and rumen microbial crude protein (MCP) metabolism, and the forage intake by nonpregnant cows decreased as follows: early lactation > mid-lactation > late lactation, pregnant cows’ lactation > dry period. For the degradation, digestion and synthesis of rumen MCP, early-lactation cows were similar to those in the mid-lactation group, but both were higher than those in the late-lactation group, while pregnant cows had greater degradation, digestion, and synthesis of MCP in the lactation period relative to the dry period. For lactating cows, especially those with calves, grazing energy requirements, methane emission metabolism and heat production were highest in August, with increased energy expenditure in winter. Overall, grazing energy, methane emissions and heat production by dry cows were low. In the context of global climate change and grassland degradation, managers must adopt different strategies according to the physiological stages of livestock to ensure a forage-livestock balance and the sustainable utilization and development of grasslands.

## 1 Introduction

Grasslands play an important role in maintaining and developing national ecological security, animal husbandry and food security (1). With improvements in living standards in China, the dietary structure of residents has changed (2), as manifested by a reduction in the consumption of food rations and a significant increase in the demand for livestock products (3). Given the tradeoffs between grazing, conservation of grassland ecosystems, and urbanization, China faces challenges in balancing forage supply and demand. Meat production per unit area of grassland in China is currently only 30% of the world average (4), while the demand for livestock products is increasing; China will need 3–12 Mha of additional pastureland between 2020 and 2050 to meet the growing demand (5). To date, natural grasslands are still the main source of forage grass (6), but grassland degradation and reduction in China have led to declines in the ecological functions of natural grasslands (7, 8), and restoration difficulties have continued to increase (9).

The Inner Mongolia region accounts for 1/4 of China’s grassland area. The carrying capacity of the grassland area exceeded 120 million SU in 2019 (the highest in China), while the supply of grassland in the Inner Mongolia region is only 50 million SU, and it is difficult for natural pasture to meet local demand. Although pasture in China has increased over the years, only Heilongjiang, Yunnan, Zhejiang, and Fujian Provinces have been able to achieve a forage-livestock balance (10). Despite having implemented ecological restoration projects, including returning farmland to grassland, rehabilitating degraded grasslands, and controlling grassland rodents (11), the Inner Mongolia region has one of the largest pasture-carrying capacity gaps and is one of the largest provinces in China (10). As a result, extensive grassland degradation and tension between livestock and grassland supply and demand have become major issues in the management of natural grasslands in the region (12, 13). Optimized decision-making is particularly important in this context and is expected to improve the forage-livestock balance. However, grassland ecosystems in northern China are driven by stochastic abiotic factors (mainly precipitation), and grassland productivity is highly unpredictable (9), which makes the spatial and temporal relationships between grasslands and livestock very complex. Grassland resources are characterized by spatial and temporal variability, and the spatial heterogeneity and temporal dynamics of forage quantity and quality are important for livestock production (14). China’s grasslands generally have abundant forage in the summer and fall but a lack of forage in the winter and spring, and the temporal variation in available forage has a much greater impact on livestock than the spatial variation in forage. If the seasonal variation is not fully considered, determining the livestock carrying capacity by estimating total forage production (11) does not effectively solve the problem of seasonal grassland overloading. The traditional total digestible nutrient (TDN) method has been shown to be difficult to adapt to most scenarios due to physiological differences among livestock, and this method has been replaced by the metabolizable energy (ME) system, which is the most effective way of balancing the nutritional requirements of livestock (15, 16).

Diagnostic techniques for determining the forage-livestock energy balance can optimize resource utilization and reduce the severe degradation of grasslands while helping to maintain the sustainable development of grassland ecosystems (17). The GRAZPLAN decision support tool, which is based on pasture and animal production models, is universally applicable for simulating biological processes in grazing systems, and it provides general prediction equations for the energy and protein requirements of all types of sheep and cattle with any physiological conditions. Alternatively, GrazFeed is a reliable tool for calculating the nutritional requirements of livestock, and by predicting food intake based on user-supplied descriptions to obtain the condition of grazing livestock, it can help ranchers implement feeding standards (18–20).

Located in the eastern part of the Inner Mongolia Autonomous Region, the Hulunbuir Grassland is one of the largest natural grasslands in the world and an important ecological barrier in China; this region is highly sensitive to climate change (21–23) and is also facing grassland degradation (24). In this study, which was informed by current local grassland livestock husbandry practices, a survey of herdsmen, and measurements of grassland productivity and livestock production, the GrazFeed and GRAZPLAN models were used to (1) investigate the energy demand of local livestock at different physiological stages and at different times and (2) explore the energy balance between grassland and livestock during the grazing period on the Hulunbuir grassland to provide a theoretical basis for resource-efficient utilization of grassland and livestock husbandry at the pasture scale.

## 2 Materials and methods

### 2.1 Overview of the study area

**Figure 1:**
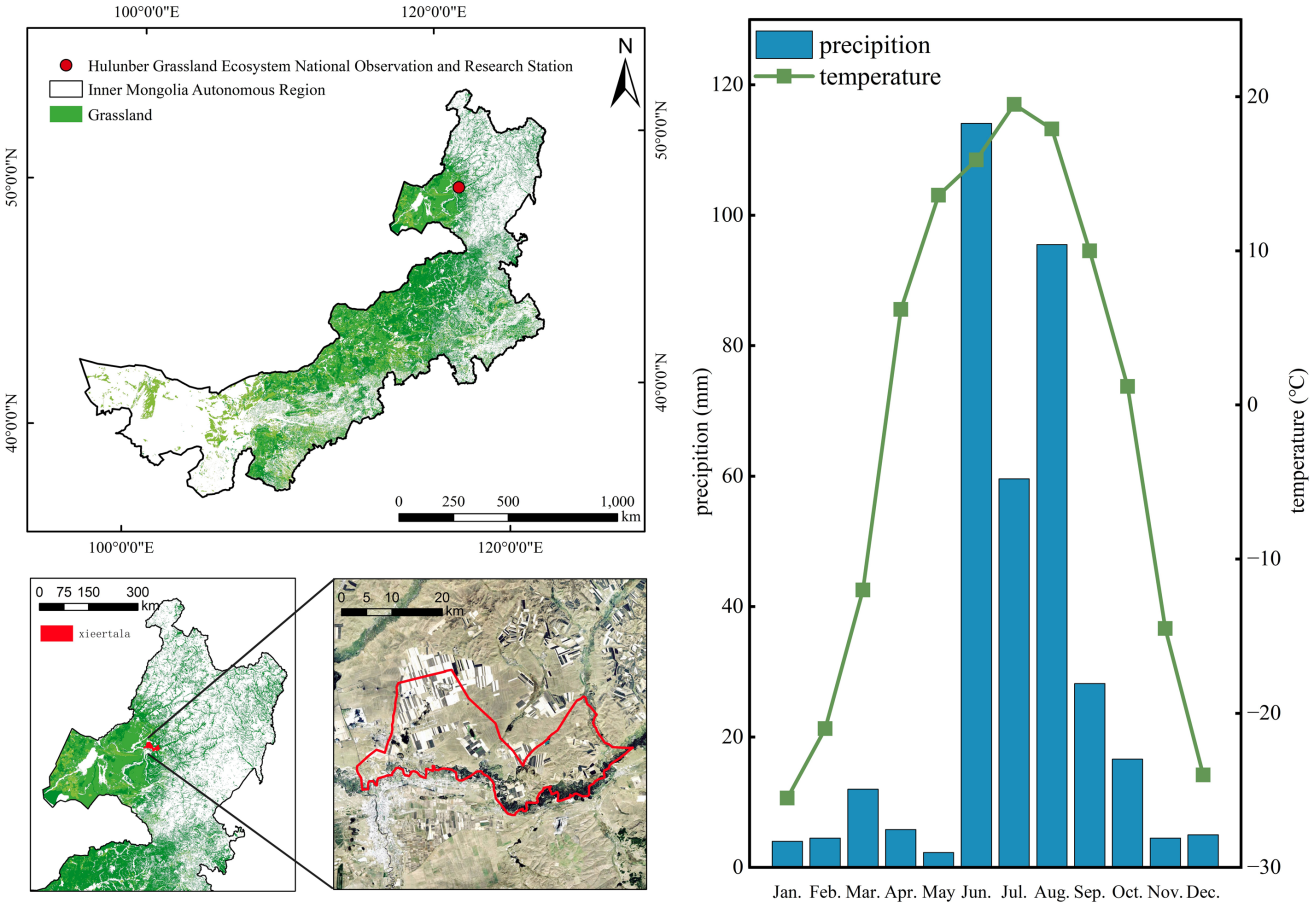
Grassland distribution in Inner Mongolia, the location of the study area, and the climate conditions.

The study area was the Xieertala Ranch, Hulunbuir City, Inner Mongolia, China (N49°19′, E120°03′, altitude 628 m). It has a temperate semiarid continental climate, with an average annual temperature of −5 to −2°C, an average annual precipitation of 350–400 mm (concentrated from July September), an annual cumulative temperature ≥10°C of 1,580–1,800°C, and a frost-free period of approximately 110 days. Topographically, the study area is an undulating high plain with black and dark chestnut calcareous soils, and the vegetation is dominated by mesic and arid plants. The representative vegetation type is temperate meadow steppe. The dominant plants include *Stipa baicalensis*, *Leymus chinensis*, *Carex pediformis*, and *Filifolium sibiricum*. The basic conditions of the study area are shown in Figure 1. Grassland resource distribution data were obtained from the Data Center for Resources and Environmental Sciences, Chinese Academy of Sciences (RESDC),^1^ and the meteorological data were obtained from the China Meteorological Data Service Centre.^2^ Xieertala Ranch is a typical state-run collective of professional joint households or medium-sized professional households with different feeding scales and different feeding methods for dairy cattle breeding. According to the current level of dairy cattle rearing in China, a decentralized household rearing mode and a state-run collective rearing mode are typical. The grazing season in the study area is relatively short. Thus, cattle ranching in the area requires mowing and supplemental feeding, and this pattern has been practiced for hundreds of years (25). In 2006, a spring grazing moratorium was introduced in the region; grazing occurs from June 1 to October 1 each year, which puts the grasslands under greater grazing pressure and causes varying degrees of degradation.

### 2.2 Research methods

#### 2.2.1 Research models

The Australian GrazFeed model with the GRAZPLAN decision support system (19, 20), in which the parameters of the model are localized based on the research of CSIRO, was used in this study. Survey data and measured data from controlled trials were incorporated into the model, and climatic conditions, grassland status, livestock status, and supplemental feeding in the study area were input for model computation and analysis.

**Figure 2:**
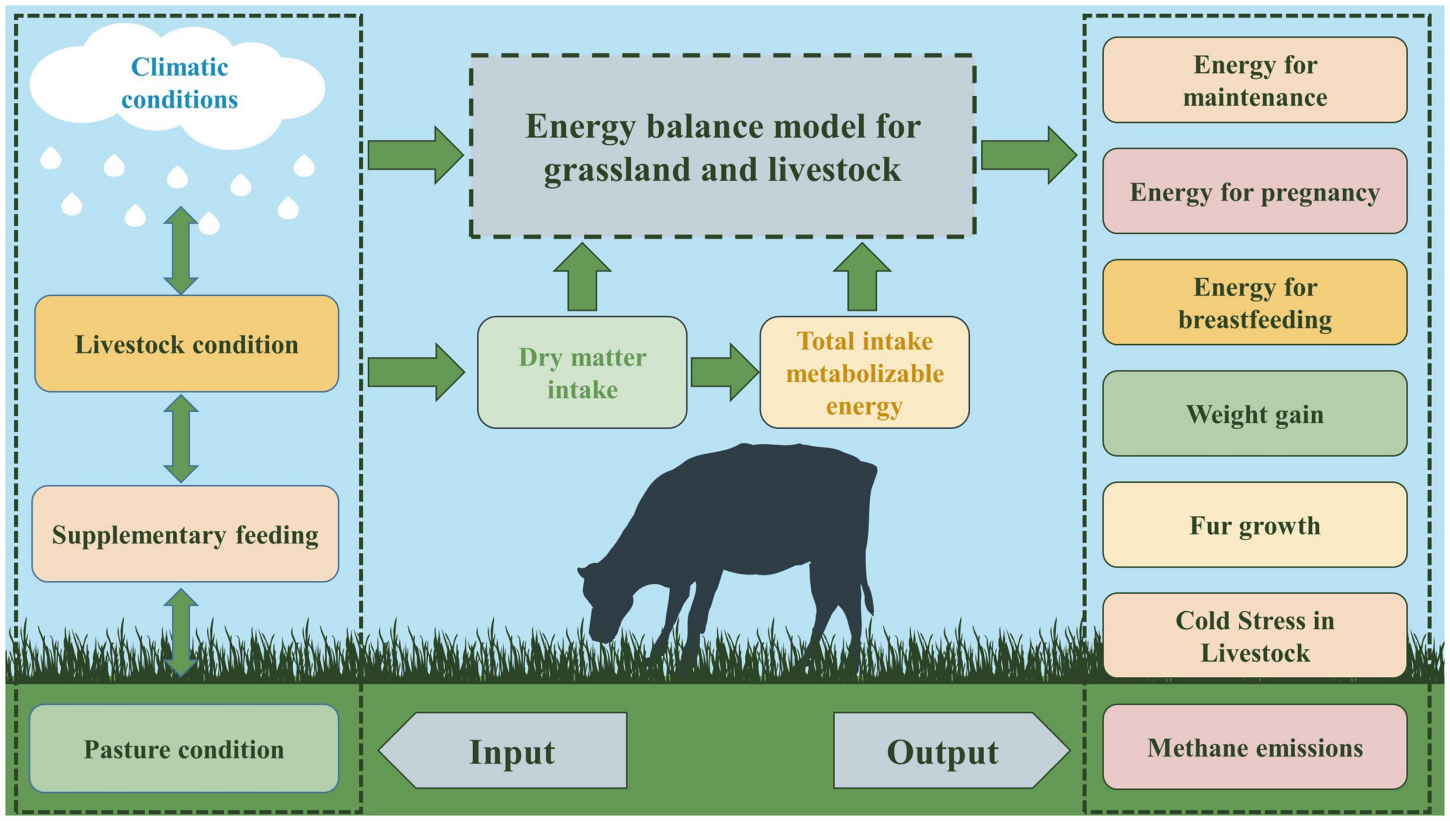
Diagnostic model of the energy balance of grasslands and livestock.

With this model, a detailed break-even analysis of the metabolic energy requirements of livestock in different months was conducted, revealing the energy balance for the local forage-livestock system. The framework of the model is shown below (Figure 2), and the specific model input parameters are shown in Table 1.

**Table 1:** Model input parameters.

Pasture conditions	Climatic conditions	Supplementary feeding	Conditions of livestock
Forage dry matter can be used	Rainfall	Dry matter ratio	Mature weight, SRW
Dry matter digestibility	Wind speed	Roughage to digestible dry matter ratio	Average net weight, W
Altitude	Maximum temperature	Metabolizable energy to dry matter ratio	Average age, Ay
Crude protein	Minimum temperature	Crude protein ratio	Body condition of livestock, BC
Month	Mean temperature	Degradable protein ratio	Average fur thickness, F
Pasture latitude	Time	Raw material price	Weight of newborn livestock, BW
Pasture slope	Day length	Supplementary feeding amount	Gestation days, Ag
Pasture area	Day of year		Body condition of livestock at the time of division, BCb
Metabolizable energy to dry matter ratio			Breastfeeding days, A
			Expected peak milk production
			Number of livestock

#### 2.2.2 Model data types

The dataset includes: (1) a grassland resource survey (grassland area, grassland type, grassland productivity and nutrient quality measurement) with data from field surveys; (2) data collection on livestock rearing by herding households (livestock weight, livestock age, livestock body condition, and livestock supplementation) with data from pasture surveys; and (3) climate data (maximum temperature, minimum temperature, average temperature, rainfall, wind speed, etc.) from the China Meteorological Data Service Centre.^3^

#### 2.2.3 Data analysis

To analyze the energy balance of cows at different time periods in the Hulunbuir region, based on the grass dynamics and climatic conditions of the Xieertala pasture, the balance between metabolizable energy (ME) and total metabolizable energy intake MEI_total_ was calculated for 500 kg adult cows at different physiological stages based on the relevant formulas and algorithms provided by the GrazFeed model and the GRAZPLAN model. In addition, we calculated the feed intake, rumen microbial crude protein (MCP), methane metabolizable energy, energy consumed by grazing, and heat production of cows at different time periods. The data were visualized using Origin 2023.

## 3 Results

### 3.1 Energy requirements of adult nonpregnant cows at different physiological stages

**Figure 3:**
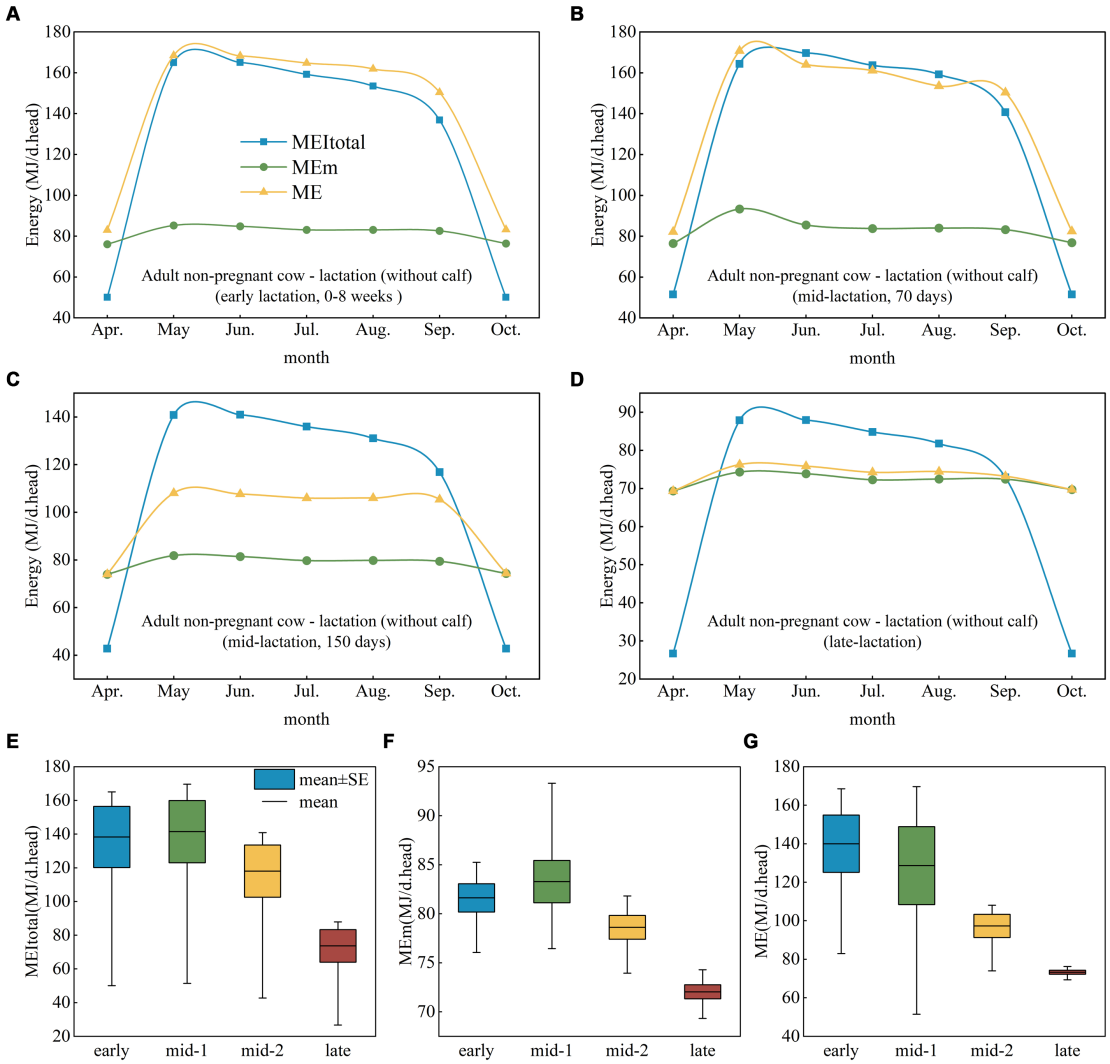
Analysis of ME, ME_m_, and MEI_total_ at different physiological stages in adult nonpregnant cows. **(A)** Early lactation. **(B)** Mid-lactation (70 days). **(C)** Mid-lactation (150 days). **(D)** Late lactation. **(E,F)** ME, ME_m_, and MEI_total_ at different physiological stages in cows. ME, metabolizable energy; ME_m_, metabolizable energy for maintenance; MEI_total_, total metabolizable energy intake.

During the grazing period from May to September, the MEI_total_ of adult nonpregnant cows in early lactation was greater than the ME_m_ (Figure 3A), but this did not meet the metabolizable energy (ME) requirement of grazing livestock. The gap between ME and the MEI_total_ of adult nonpregnant cows in early lactation was small in late spring (May–June) and widened in July. Adult nonpregnant cows in mid-lactation (70 days) had a greater MEI_total_ than ME in the summer (June–August) to meet livestock requirements, but they were still unable to meet their ME_m_ from September to May (Figure 3B). During the mid-lactation period (150 days), the ME_m_ and ME requirements could be satisfied from April to October (Figure 3C). Although the MEI_total_ of adult nonpregnant cows decreased significantly in late lactation (Figure 3E), it could still meet the ME and ME_m_ requirements of livestock from May to August, and ME was almost equal to the ME_m_ (Figure 3D). However, the MEI_total_ of nonpregnant adult cows at different physiological stages from October to April could not meet the ME_m_ or ME needs.

The energy requirements of adult nonpregnant cows varied considerably at different physiological stages; the MEI_total_ of cows was essentially the same in early lactation and in mid-lactation (70 days) and then decreased in mid-lactation (150 days) and in late lactation (Figure 3E). The ME_m_ requirement of adult nonpregnant cows reached its maximum at mid-lactation (70 days), but it also fluctuated greatly (Figure 3F). In adult nonpregnant cows, ME needs decreased from early lactation to late lactation, and energy requirements fluctuated the most at mid-lactation (70 days) (Figure 3G).

### 3.2 Energy requirements of pregnant adult cows at different physiological stages

**Figure 4:**
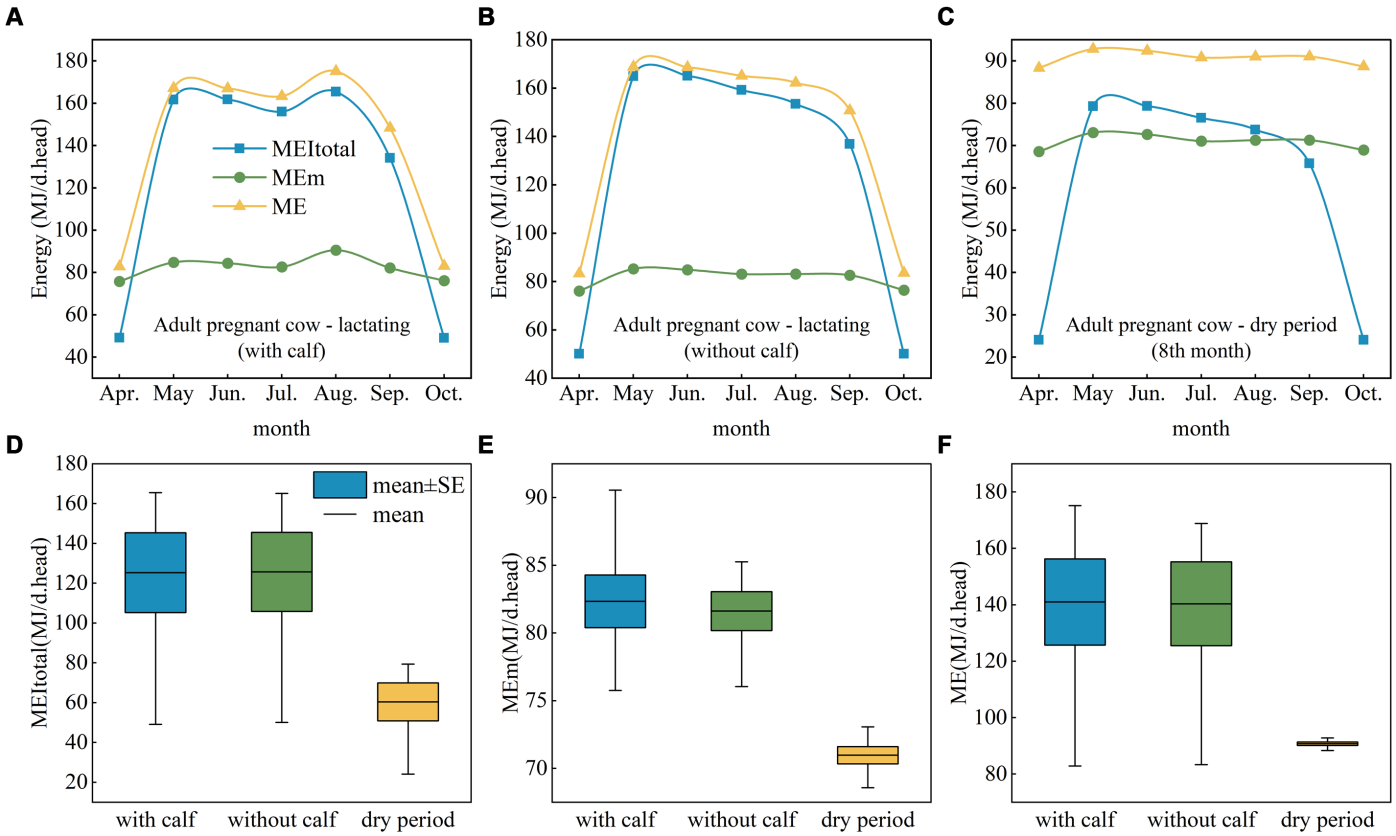
ME, ME_m_, and MEI_total_ for adult pregnant cows at different physiological stages. **(A)** Adult pregnant lactating cow (with calf). **(B)** Adult pregnant lactating cow (without calf). **(C)** Adult pregnant dry cow. **(D–F)** ME, ME_m_, and MEI_total_ for adult pregnant lactating and dry cows. ME, metabolizable energy; ME_m_, metabolizable energy for maintenance; MEI_total_, total metabolizable energy intake.

During the grazing period (May to September), the MEI_total_ of adult pregnant lactating cows with and without calves was greater than ME_m_, and the ME requirement of grazing livestock could not be met (Figures 4A–C), while the MEI_total_ of pregnant dry cows could not meet the ME_m_ requirement even in September (Figure 4C). For pregnant cows with calves, MEI_total_ and ME increased in August, but for cows without calves and dry cows, MEI_total_ and ME decreased after May (Figures 4B,C). The MEI_total_, ME and ME_m_ of adult pregnant cows with or without calves were similar but were much greater than those of dry cows (Figures 4D–F). This study revealed the same trend for adult pregnant cows and adult nonpregnant cows from October to April, indicating that MEI_total_ could not meet ME_m_ or ME needs.

### 3.3 Forage dry matter intake at different physiological stages in adult cows

**Figure 5:**
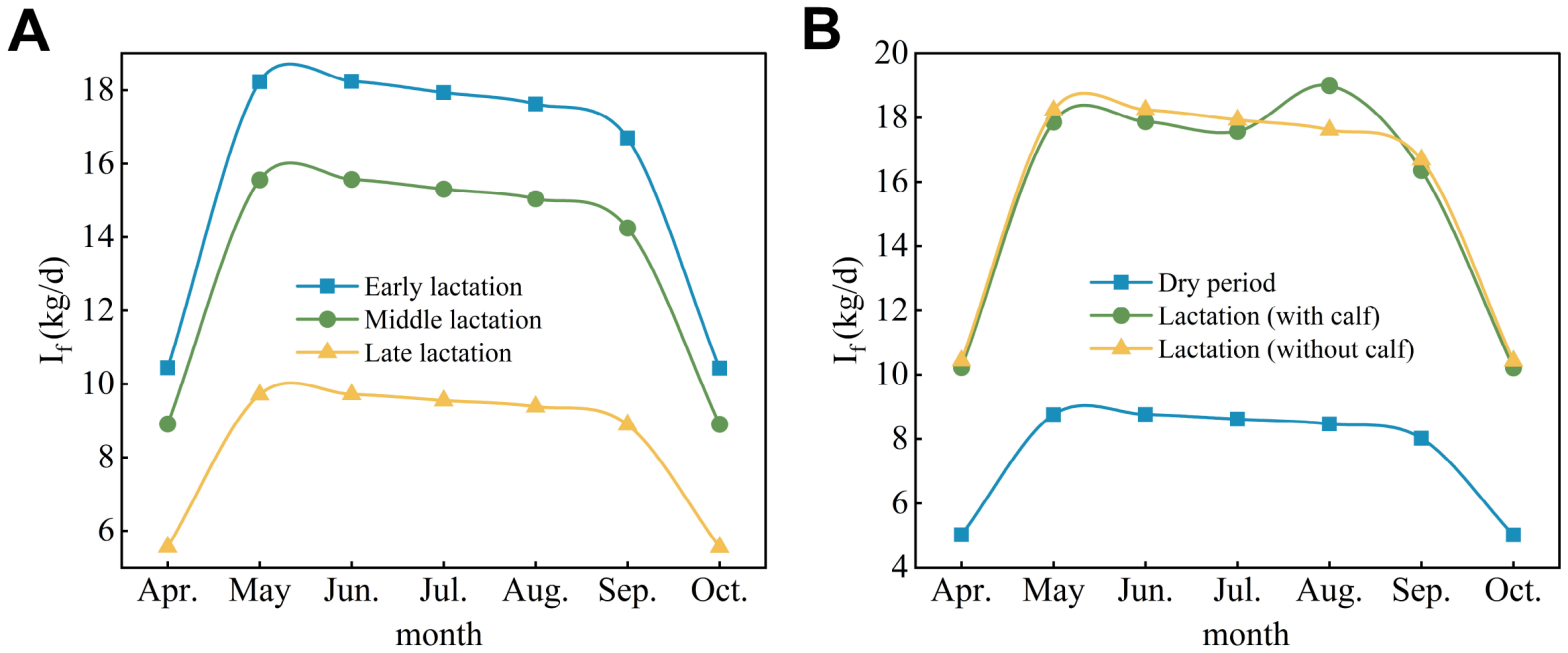
Forage dry matter intake by adult cows at different physiological stages. **(A)** Nonpregnant lactating cows (without calf). **(B)** Pregnant cows. I_f_, feed intake.

Adult cows at different physiological stages exhibited different forage dry matter intake characteristics. Specifically, forage dry matter intake by nonpregnant cows decreased as follows: early lactation > mid-lactation > late lactation (Figure 5A), and the range of forage dry matter intake was 10.43–18.24 kg DM/d, 8.90–15.57 kg DM/d, and 5.55–9.71 kg DM/d during the early lactation, mid-lactation and late lactation periods, respectively. Forage dry matter intake by pregnant cows was greater during lactation than during the dry period (Figure 5B). During lactation, the forage dry matter intake ranged from 10.22–18.99 kg DM/d from April–October, while during the dry period, forage dry matter intake ranged from 5.00–8.76 kg DM/d from April–October.

### 3.4 MCP degradation, digestion, and synthesis at different physiological stages in adult cows

**Figure 6:**
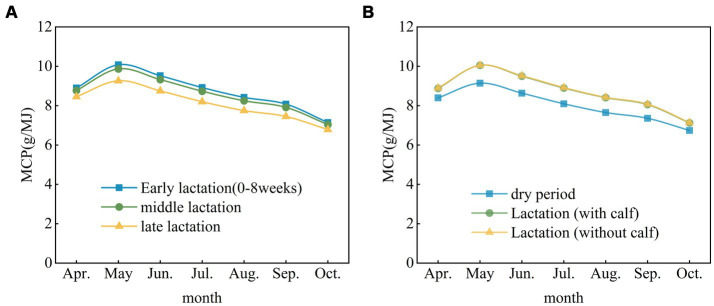
Degradation, digestion and synthesis of MCP at different physiological stages in adult cows. **(A)** Adult nonpregnant cows. **(B)** Adult pregnant cows. MCP, rumen microbial crude protein.

The degradation, digestion and synthesis of rumen MCP in nonpregnant cows and adult pregnant cows were similar in early lactation and mid-lactation and were greater than they were in late lactation (Figure 6A). The degradation, digestion and synthesis of MCP by pregnant lactating cows with calves and pregnant lactating cows without calves were similar. Overall, the degradation, digestion and synthesis of MCP by pregnant cows were greater during lactation than during the dry period (Figure 6B).

### 3.5 Grazing, methane metabolism, and energy requirements of adult cows at different physiological stages of life

**Figure 7:**
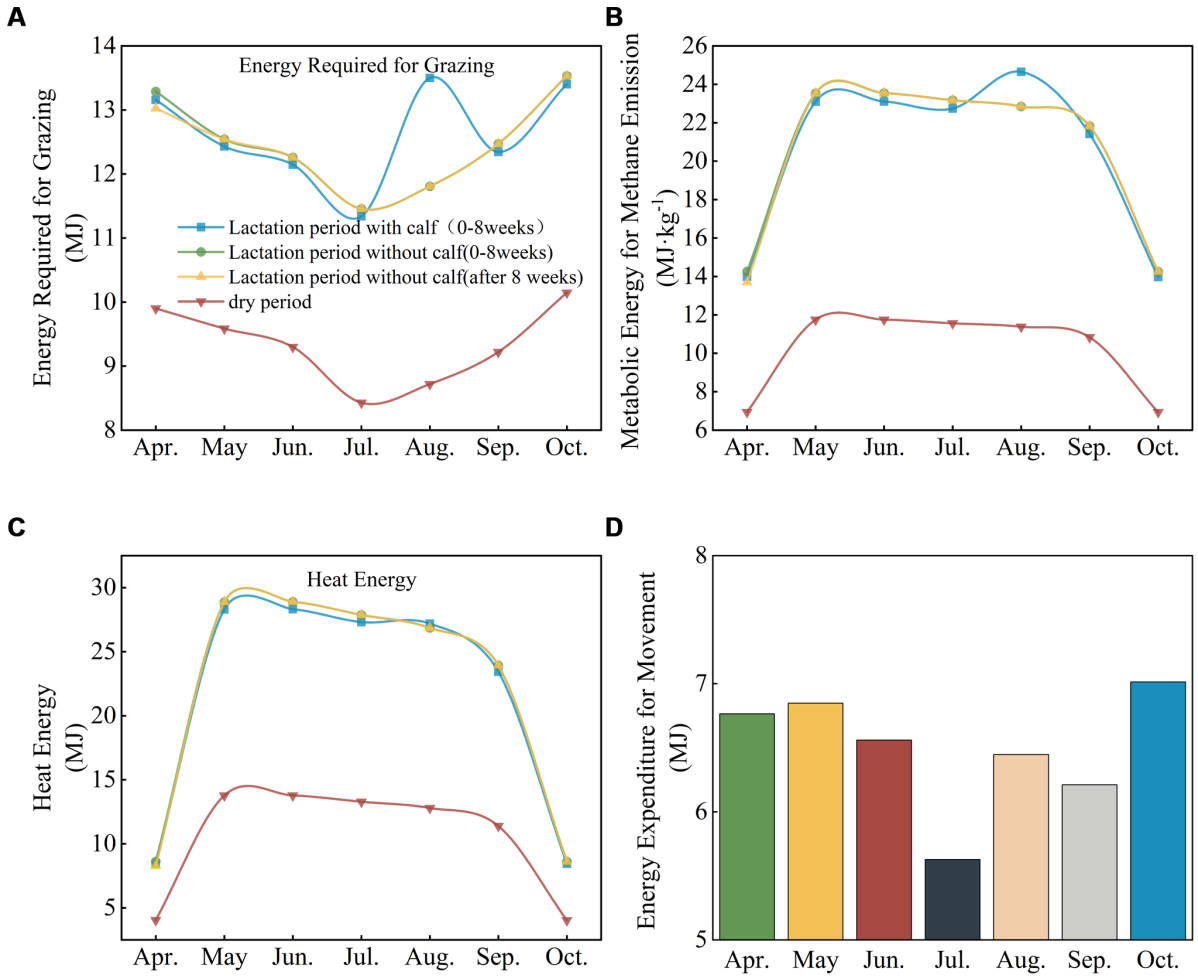
Grazing, methane metabolism and energy requirements of adult cows at different physiological stages. **(A)** Energy required for grazing by adult cows at different physiological stages and grazing periods. **(B)** Energy required for methane metabolism at different physiological stages and grazing periods. **(C)** Energy required for heat production (HP) at different physiological stages and grazing periods. **(D)** Energy required for movement at different physiological stages and grazing periods.

Adult lactating cows with calves had higher grazing energy requirements and higher metabolizable methane emissions in August than did adult cows without calves. In other months, adult lactating cows with or without calves had similar grazing energy requirements, methane emission metabolism, and heat production. Dry adult cows had lower grazing energy requirements, lower metabolizable energy for methane emissions, and lower heat production (HP) than lactating cows. In addition, the energy consumed by moving cows was lower in summer and higher in spring, fall and winter (see Figure 7).

## 4 Discussion

### 4.1 Energy requirements of nonpregnant cows at different physiological stages

The MEI_total_ of adult nonpregnant cows was lower than the ME requirement during early lactation, higher than the ME requirement during the peak pasture season in early lactation, and it remained higher than the ME requirement during most of the pasture season in late lactation, which indicates that as lactation ended, cows gradually transitioned from not being able to meet their ME needs to being able to meet their ME needs. The results showed that the MEI_total_ of beef cows gradually transitioned from not meeting the ME requirement to meeting the ME requirement as lactation ended. This transition was not due to a change in MEI_total_ during lactation but to a decrease in the ME requirement as lactation ended and to ME reaching a level similar to ME_m_. The physiological characteristics of adult cows lead to changes in ME and ME_m_ needs at different stages (26). Cows in early lactation and mid-lactation must meet the demands of high milk production, which creates an increase in the ME requirement (27). In particular, the ME requirement is highest at approximately 70°C during lactation and fluctuates greatly, which may result in MEI_total_ not being able to meet the ME requirement; therefore, forage supplementation must be considered. Late lactating cows are still able to meet their metabolic needs despite a decrease in MEI_total_ during the summer and therefore winter supplemental feeding should be considered.

Seasonal resource variation is an important cause of the imbalance between the energy intake and demand of cows. Seasonal changes in grasslands directly affect the supply and nutritional content of forage, thus impacting energy intake by beef cattle. In this study, a serious decline in MEI_total_ by adult nonpregnant cows grazing from late fall to early spring was observed; the MEI_total_ of cows during this period was already lower than the ME_m_ requirement, which would cause weight loss if forage was not supplemented. Seasonal factors in grassland ecosystems directly affect forage availability (28). More grass growth occurs in the summer, making it easier for cows to meet their energy needs at this time of year (29). Restricted grassland resources in winter result in insufficient MEI_total_. Therefore, local ranchers must balance energy supply and maintenance by supplementation during different reproductive periods (30). For example, managers may feed early lactating cows more to meet their higher energy requirements or adjust their reproductive cycle so that early lactation occurs as close as possible to the peak forage season.

### 4.2 Energy demand and supply for pregnant cows at different physiological stages

We found that the MEI_total_ of gestating cows would not meet the ME requirement throughout the year if cows relied only on grazing; it was even difficult for cows to meet their ME_m_ needs from October to April. Therefore supplemental feeding at the beginning and the end of the grazing season for gestating cows must be considered to prevent weight loss. For pregnant dry cows, there was a large gap between MEI_total_ and ME needs at all times of the year, and MEI_total_ did not meet the ME_m_ requirement during the three months of the grazing season. Therefore, in addition to supplemental feeding at the beginning and end of the grazing period, supplemental feeding during other grazing periods is also desirable.

The MEI_total_ of pregnant dry cows cannot meet the ME requirement because MEI_total_ is too low-energy in this period. In addition, depletion of the protein stored in the body during the dry period has a negative impact on health during subsequent reproduction and lactation. Managers must pay more attention to the nutritional intake of dry cows and select appropriate diets for supplementation. Preperinatal diets should contain more metabolizable protein and energy than early dry period diets, but energy and fiber should be controlled to ensure adequate intake after calving (31). However, studies have shown that reducing the duration of the dry period did not affect the health or fertility of livestock, which means that reducing the duration of the dry period may be conducive to the efficient use of pasture resources (32).

### 4.3 Changes in feed intake at different physiological stages in cows

Feed intake, digestion and energy requirements changed during different physiological stages. Studies have shown that for nonpregnant cows, forage dry matter intake is greater in early lactation than in other periods, and these cows require more energy to support milk production (33); as a result, forage dry matter intake is greater. This is related to the need for more energy during early lactation to cope with physiological preparations for lactation and early milk production, which means that early in the lactation cycle, more energy-rich forage may be required to ensure proper nourishment, performance, and health during the production cycle. Similarly, forage dry matter intake is greater for pregnant cows during lactation because extra energy is required to support fetal production during pregnancy. Managers may need to provide more abundant feed to ensure productivity. In addition, seasonal resource variability plays a key role in forage intake (34). From May to September, grassland resources are abundant and vegetation grows rapidly. The actual forage intake by cows during this period is high, especially for early-lactation and mid-lactation cows. However, from October to April, grass resources become limited, and forage availability decreases, resulting in a significant reduction in forage intake by cows. This seasonal change in resources directly affects the energy intake of cows. The physiological status of cows also affects their forage intake. Lactating cows need to meet high milk production and reproduction demands, so their energy requirements are higher; accordingly, their forage intake on pasture is also greater. In contrast, dry cows have lower energy requirements and a lower forage intake.

### 4.4 Changes in the degradation, digestion and synthesis of MCP by cows at different physiological stages

Changes in MCP degradation and digestion by cows at different physiological stages have important effects on energy intake and metabolism. We showed that the degradation, digestion and synthesis of MCP were relatively stable during early lactation and mid-lactation; however, the degradation, digestion and synthesis of MCP decreased during late lactation, which implied that cows required more easily degraded and absorbed feed at this physiological stage to meet their energy requirements. Late lactating cows are often challenged with regaining postpartum weight and beginning a new round of pregnancy, so they need higher energy levels to maintain body function and support pregnancy. In livestock management, understanding the energy requirements and digestive characteristics of cows at different physiological stages is critical for developing appropriate feeding strategies. This includes the selection of appropriate forage types and feeding practices to ensure that cows receive the energy they need while maintaining the sustainability of the farm.

### 4.5 Changes in grazing, methane metabolism, and energy requirements of cows at different physiological stages

Studies have shown that cows require more metabolizable energy from methane during the summer months and that the methane produced in the gastrointestinal tract of ruminant livestock represents an important loss in the body’s energy utilization process; it accounts for 10% of the total energy from feedstuffs and 87–89% of intestinal methane production comes from the rumen (35). Related studies have shown a positive correlation between methane production and livestock feed intake (36). Methane is an important greenhouse gas, and reducing methane emissions from livestock can reduce both the greenhouse effect and energy losses from livestock. High-starch grain-based diets immediately reduce methane emissions, whereas forage-based diets cause an increase in methane emissions (35). In the northern grasslands of China, the most vigorous pasture growth occurs in August; therefore, livestock forage more during this period, and higher feed intake explains methane metabolism energy and feed intake changes (37). Frequent feeding under grazing conditions also requires more energy, while limited pasture resources at the beginning and end of the grazing period result in greater movement of livestock, which further increases their energy consumption (29).

Modeling results indicate that heat production by cows is greater in the summer than in the spring or fall during the grazing period because ruminants are active during the day and sleeping or inactive at night, and more heat is produced during the day than during the night (38). After feeding, rumen fermentation and nutrient absorption in the digestive tract lead to the accumulation of large amounts of metabolic heat (39). When ambient temperature is close to mammalian body temperature, the only viable pathway for heat loss is evaporation; if ambient temperature exceeds body temperature, heat flow is reversed, the animal shifts to a heat-shedding phase, and domestic animals expend more energy to maintain their body temperature (40). Because cattle are more cold-tolerant, their thermoregulatory mechanisms help them maintain homeostasis under wintry conditions. Heat production is relatively low in winter, especially in dry, cold climates, and it is relatively easy for animals to maintain their heat balance (41).

### 4.6 The effectiveness and limitations of forage-livestock balance models

The methods of assessing forage-livestock balance remain controversial (42, 43), but contemporary scientists no longer rely solely on the simplistic theory of equilibrium for calculating this balance (44). To understand the relationship between forage management and livestock production, modeling (45) and forage supply curves (46) are being used to evaluate the forage-livestock balance of grasslands. However, these studies have failed to adequately capture the impact of climate change on forage supply. To account for livestock adaptation to grasslands and the influence of abiotic factors, scientists have utilized remote sensing models to monitor aboveground biomass and assess forage-livestock balance over extensive grassland regions (42), but this approach is limited to evaluating grazing potential across regions and cannot be used to assess the status of livestock during various grazing periods. Only dynamic simulation models can capture complex interactions between livestock and feed supply dynamics over time, although these models are highly intricate and require extensive input data (47). These model improve our understanding of seasonal variation in livestock metabolic energy requirements and the imbalance between supply and demand in Hulunbuir, and they provide effective management strategies for local farmers and herdsmen that can improve production efficiency and promote the sustainable management of grasslands in the context of changing seasonal environments. Although the present study integrated multiple data points to assess the metabolic energy demand and intake of local livestock in Hulunbuir, the accuracy and practicality of the model may still be improved. First, data collection can be improved by including livestock growth performance and grassland quality data. Data collection was limited by survey methods that had difficulty comprehensively covering the Hulunbuir region, which may have introduced bias to the results. Second, models may need more refinement and better parameterization to reflect livestock needs under different local conditions, which may require more observations. Lastly, model application may be constrained by the scope and quality of available data, as well as the specific region where the model is used. This limitation could affect the accuracy and applicability of the model results. More comprehensive data collection and validation, along with further optimization and validation of the model, will support the implementation of efficient and sustainable grassland-livestock management decisions in the Hulunbuir region.

## 5 Conclusion

The MEI_total_ of adult cows at different physiological stages was greater than the ME_m_ requirement in late spring-early fall of the grazing period. The MEI_total_ of early lactating cows was lower than the ME requirement, while the MEI_total_ in the middle and late lactation periods was greater than the ME requirement. The MEI_total_ of adult pregnant cows did not meet ME needs. From October to April, this study revealed that the MEI_total_ of adult cows was significantly lower than the ME_m_ and ME needs, and there was an imbalance between energy supply and demand.

The ME requirement and MEI_total_ for nonpregnant cows, the intake of forage dry matter, and the degradation, digestion and synthesis of rumen MCP all decreased gradually from early lactation to middle lactation to late lactation, and the lactation period was longer than the dry period for pregnant cows.

Pregnant adult lactating cows had a MEI_total_ that was greater than the ME_m_ requirement and lower the ME requirement during the grazing period from late spring to early fall (May–September). During the dry period, the MEI_total_, feed intake, MCP degradation, digestion and synthesis, grazing energy consumption, methane metabolism energy and HP of pregnant cows decreased. This study will improve our responses to climate and resource changes in the Hulunbuir grassland and contribute to sustainable grassland ecosystem management and livestock production.

## Data availability statement

The original contributions presented in the study are included in the article/supplementary material, further inquiries can be directed to the corresponding authors.

## Author contributions

TY: Methodology, Writing – original draft. RY: Data curation, Methodology, Supervision, Writing – review & editing. XX: Conceptualization, Data curation, Methodology, Writing – review & editing. XZ: Supervision, Validation, Writing – review & editing. GY: Methodology, Supervision, Writing – review & editing.

## Footnotes

^1^ http://www.resdc.cn

^2^ https://data.cma.cn/

^3^ https://data.cma.cn/
