# Corrigendum: Simulation of the nutritional requirements and energy balance of adult cows in a northern temperate grassland

**DOI:** 10.3389/fvets.2024.1531293

**Published:** 2024-12-05

**Authors:** Tianqi Yu, Ruirui Yan, Xiaoping Xin, Xiaoying Zhang, Guomei Yin

**Affiliations:** ^1^State Key Laboratory of Efficient Utilization of Arid and Semi-Arid Arable Land in Northern China, Institute of Agricultural Resources and Regional Planning, Chinese Academy of Agricultural Sciences, Beijing, China; ^2^Hulun Buir Agricultural Technology Extension Center, Hailar, China; ^3^Grassland Research Institute of Inner Mongolia Academy of Agricultural & Animal Husbandry Sciences, Hohhot, China

**Keywords:** pasture management, energy balance, Hulunbuir grassland, forage-livestock balance, livestock management

In the published article, there was an error in Figure 5 as published.

Figure 5 was a duplicate of Figure 6. The corrected figure and legend appear below.

**Figure 5:**
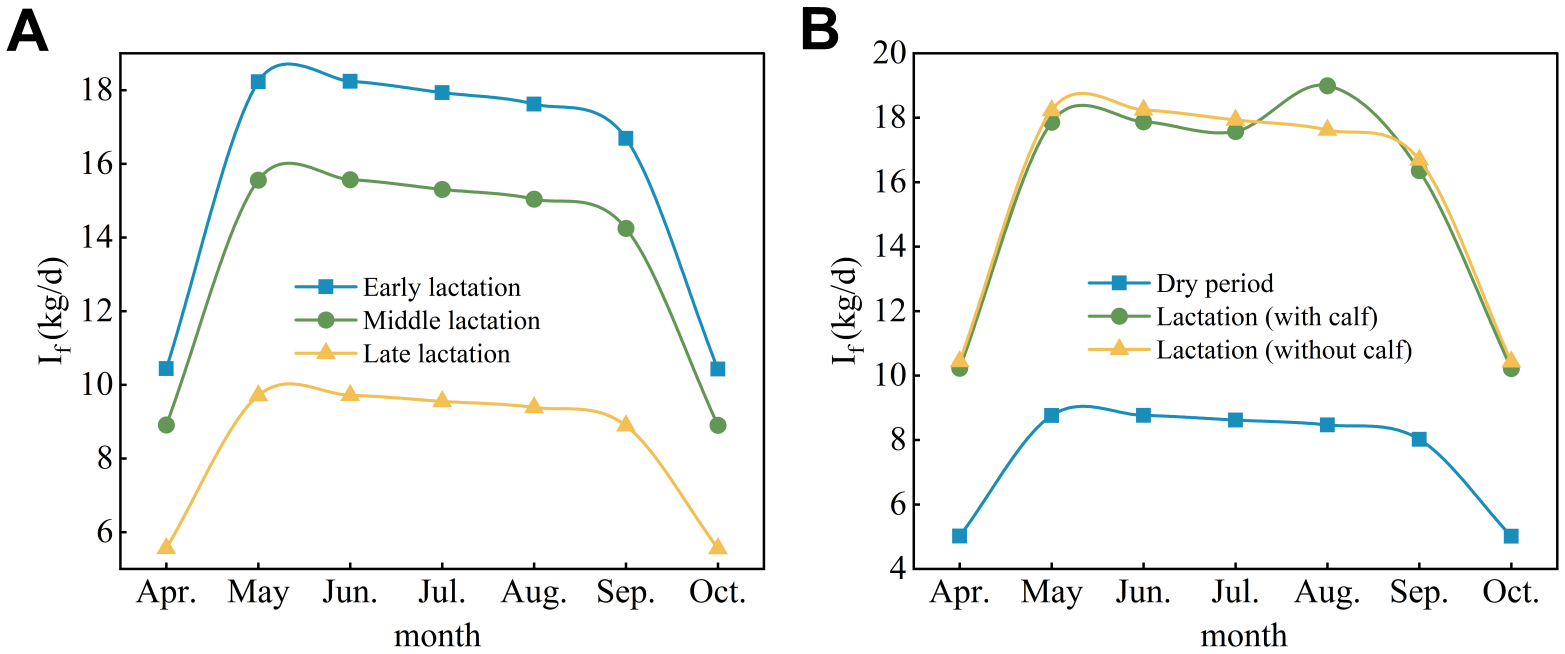
Forage dry matter intake by adult cows at different physiological stages. **(A)** Nonpregnant lactating cows (without calf). **(B)** Pregnant cows. I_f_, feed intake.

The authors apologize for this error and state that this does not change the scientific conclusions of the article in any way. The original article has been updated.

